# Population-Level Impact of the Enterovirus A71 Vaccination Program on Hand, Foot, and Mouth Disease: Ecological Time-Series Study

**DOI:** 10.2196/85604

**Published:** 2026-03-10

**Authors:** Ye Tong, Huan Fan, Jing Wang, Xinru Zeng, Xiaoqing Cheng, Changjun Bao, Liguo Zhu, Hong Ji, Xiang Huo

**Affiliations:** 1 Department of Acute Infectious Disease Control and Prevention, Jiangsu Provincial Center for Disease Control and Prevention, 172 Jiangsu Road, Nanjing, 210009, China, 86 25-83759421; 2 School of Public Health, Nanjing Medical University, Nanjing, China

**Keywords:** hand, foot, and mouth disease, enterovirus A71, vaccine efficacy, Chinese population, counterfactual model, real-world study

## Abstract

**Background:**

Hand, foot, and mouth disease (HFMD), a common childhood illness caused by various enteroviruses, poses a significant public health threat in the Asia-Pacific region, where severe cases associated with enterovirus A71 (EV71) are a major concern. The EV71 vaccination program was introduced in China in late 2016. Although randomized controlled trials have established the robust efficacy and safety of these vaccines, assessing their real-world performance remains crucial. Subsequent studies have evaluated its real-world effectiveness in several provinces, including Zhejiang and Guangdong. However, evidence on its real-world impact in reducing EV71-associated HFMD in Jiangsu Province remains limited.

**Objective:**

This study aimed to describe HFMD epidemiological characteristics and to evaluate the population-level effectiveness of the EV71 vaccination program in Jiangsu Province.

**Methods:**

We characterized the temporal distribution of EV71-related HFMD cases based on longitudinal surveillance data collected through the National Notifiable Diseases Surveillance System in Jiangsu Province from 2012 to 2019 and assessed the impact of vaccination using a Bayesian structural time series model under a counterfactual framework. The intervention effect of the EV71 vaccine was estimated by comparing the observed data with predictions from a counterfactual model scenario without vaccination.

**Results:**

A total of 932,274 HFMD cases were reported in Jiangsu from 2012 to 2019, including 5190 (0.56%) severe cases. An estimated 140,876 (15.11%) cases were attributed to EV71. EV71-associated HFMD cases showed a marked decline, with estimated numbers of 22,303, 9796, and 3900 in 2017, 2018, and 2019, respectively. We estimated that 30,117 EV71 cases (95% credible interval −1777 to 93,601) were prevented by the vaccination program from 2017 to 2019, corresponding to a reduction of 45.55% (95% credible interval −5.19% to 72.22%). The effectiveness of vaccination increased annually, with reductions of −1.03% (95% credible interval −94.85% to 48.29%) in 2017, 55.54% (95% credible interval 13.94%-77.56%) in 2018, and 82.28% (95% credible interval 65.77%-90.89%) in 2019. Furthermore, we observed that children younger than 4 years obtained greater benefits compared with those older than 4 years, with the greatest reduction of 57.68% (95% credible interval 13.04%-79.65%) in the 3- to 4-year age group, followed by a 48.09% (95% credible interval 28.00%-62.98%) reduction in the 0- to 2-year age group. In contrast, the reduction was markedly lower, at 16.75 % (95% credible interval −96.53% to 65.47%) in children older than 4 years during the 2017 to 2019 period.

**Conclusions:**

EV71 vaccination is an effective measure to prevent HFMD. The sharp decline in EV71-associated HFMD cases following the implementation of the EV71 vaccination program suggests a potential causal relationship. Therefore, strengthening vaccine coverage remains a public health priority.

## Introduction

Hand, foot, and mouth disease (HFMD) is a highly contagious viral illness that is endemic to China and poses a significant public health burden, particularly among children younger than 5 years [1]. Surveillance data from the Chinese Center for Disease Control and Prevention identified 7,200,092 HFMD cases from 2008 to 2012, resulting in 2457 deaths [2]. HFMD can be caused by various enteroviruses, such as enterovirus A71 (EV71) and coxsackieviruses, including serotypes A16, A6, A10, B1, B2, and others. Among the various enteroviruses responsible for HFMD, EV71 has been identified as a major pathogen associated with severe and fatal cases [3]. Its neurotropic nature allows it to cross the blood-brain barrier, potentially causing serious neurological complications, such as poliomyelitis-like paralysis, encephalitis, and fatal cardiopulmonary failure in young children [4,5]. Moreover, EV71 is highly contagious and can be transmitted directly via respiratory droplets, saliva, or the fecal-oral route [6], as well as indirectly through fomites on various surfaces such as metal, plastic, and paper [7].

Epidemiological surveillance data have highlighted the substantial disease burden caused by EV71. In 2016, EV71 was estimated to be responsible for approximately 70% of severe HFMD cases and more than 90% of HFMD-associated deaths in China [8,9]. In response to this threat, several inactivated EV71 vaccines were developed by Chinese pharmaceutical companies and were granted regulatory approval in 2015 for use in preventing EV71-related HFMD [10-13]. The national EV71 vaccination program was subsequently launched in 2016, with large-scale promotion and mobilization efforts taking place from 2017 onward [14].

While randomized controlled trials [11-13] have demonstrated the high efficacy and safety of these vaccines in controlled clinical settings [15], it is essential to evaluate their performance under real-world conditions. Postmarketing surveillance and population-level evaluations provide critical evidence to guide public health policy and vaccination strategies. A few recent studies have provided encouraging real-world data. For instance, Xiao et al [16] reported a 41.4% (95% credible interval 37.1%-43.7%) reduction in EV71-associated HFMD cases in Guangdong Province between 2017 and 2019 following the introduction of the vaccine. Similarly, Zheng et al [17] observed a 29% (95% credible interval 24%-34%) decline in EV71-related HFMD cases in Zhejiang Province. Additionally, research by Head et al [18] reported a 60% (95% credible interval 41%-72%) reduction in Chengdu City of Sichuan Province between 2017 and 2018. Recently, a nationwide study further estimated that EV71 vaccination was associated with a reduction of approximately 297,946 EV71-related cases during 2017 to 2018, corresponding to an overall decline of nearly 17% [19]. However, the effectiveness of the EV71 vaccination program varies across regions in China, exhibiting considerable spatial heterogeneity that warrants further investigation.

Despite these findings, there remains a paucity of evidence from other key regions in China. Jiangsu Province, located in eastern China, is one of the most densely populated and economically developed provinces in the country, with high mobility and urbanization that could influence disease transmission dynamics [20]. However, the population-level effectiveness of the EV71 vaccination program in Jiangsu has not been thoroughly investigated.

In this study, we aimed to generate population-level evidence on the real-world effectiveness of the EV71 vaccination program in Jiangsu Province. By leveraging high-quality, longitudinal surveillance data and using an advanced Bayesian structural time series (BSTS) model, we sought to quantify the vaccine’s impact on EV71-associated HFMD and provide insights to inform future vaccination strategies and public health decision-making in other endemic regions.

## Methods

### Study Population

**Figure 1.**
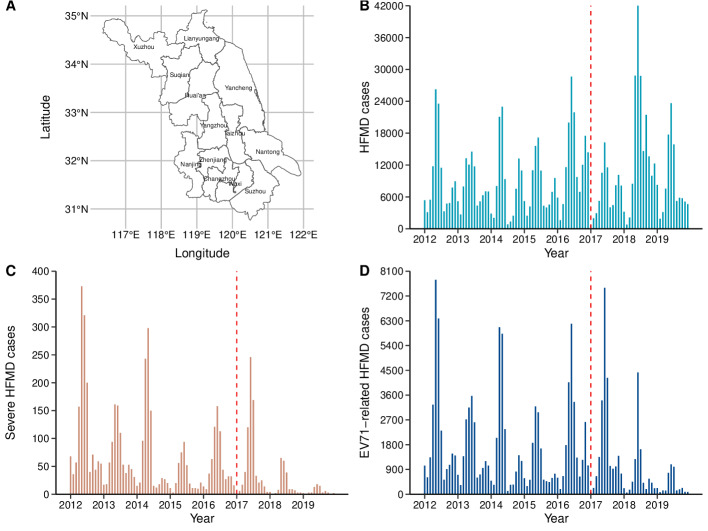
The red dotted line denotes the initiation of large-scale promotion and mobilization for vaccination. (A) Study area comprising 13 cities in Jiangsu Province, China; (B) temporal trends in total hand, foot, and mouth disease (HFMD) cases; (C) severe HFMD cases; and (D) enterovirus A71 (EV71)–related HFMD cases.

We conducted this longitudinal observational study in Jiangsu, located in eastern China, which comprises 13 cities, such as Nanjing, Suzhou, and Changzhou (Figure 1A). This study followed the STROBE (Strengthening the Reporting of Observational Studies in Epidemiology) checklist (Checklist 1). The study population consisted of children aged 0 to 14 years, totaling approximately 11 million in 2017 [21].

### Data Collection of HFMD Surveillance Data

We included reported cases of HFMD among children aged 0 to 14 years reported between 2012 and 2019. HFMD is a notifiable infectious disease in China. Clinically diagnosed cases are required to be reported through the National Notifiable Diseases Surveillance System, a nationwide direct network reporting platform. Demographic and diagnostic information for each HFMD case is included in the report. Physicians must complete and submit a case report card within 24 hours of diagnosis. The information on the report card is subsequently reviewed by the Centers for Disease Control and Prevention at the county, municipal, and provincial levels.

Etiological data on HFMD cases from 2012 to 2019 were obtained from the HFMD etiological surveillance system in Jiangsu Province. According to the surveillance protocol, each county-level hospital was required to collect specimens from at least 5 first-time medical visits for HFMD each month; if fewer than 5 cases were reported, specimens from all available cases were collected whenever possible. In the event of an HFMD outbreak, a minimum of 5 specimens were required for pathogen testing. Additionally, all severe and fatal cases were mandatorily sampled.

Specimens were tested for EV71, coxsackievirus A16, and other enteroviruses using polymerase chain reaction for nucleic acid detection. Because not all reported HFMD cases underwent etiological testing, we used a statistical method to estimate the monthly number of EV71-related HFMD cases:

EV71 cases = (number of general HFMD cases × EV71-positive rate among general cases) + number of EV71-positive cases among severe HFMD cases.

General cases were defined as fever accompanied by rashes on the hands, feet, mouth, or buttocks (with some cases presenting without fever), while severe cases were defined as those with neurological involvement or respiratory or circulatory dysfunction.

### Statistical Analysis

Within a counterfactual framework, we used a BSTS model to evaluate the intervention effects of the EV71 vaccination. The BSTS model is a sophisticated state-space model that integrates Bayesian inference to analyze time-series data [22,23]. It decomposes a time series into interpretable components, such as trend, seasonality, and external covariates, while also providing not only point predictions but also credible intervals, making it a versatile tool in epidemiology.

First, we developed BSTS models based on training sets including EV71-induced cases from 2012 to 2016. Under the counterfactual scenario assuming the absence of the EV71 vaccination program, the model was developed to simulate the monthly number of EV71-related cases. The BSTS model was defined as follows:


(1) $$log\left(y_{t}\right)=Z_{t}^{T}\alpha_{t}+\varepsilon_{t}$$

(2) $$\alpha_{\text{t+}1}=T_{t}\alpha_{t}+R_{t}\eta_{t}$$

The equation (1) was the observation equation, which related the logarithmically transformed EV71-induced monthly incidence $y_{t}$ to a vector of latent “state” variables *$a_{t}$*. In our study, the $a_{t}$ contained long-term trend (year) and seasonality (12 months each year). The transition equation (2) specifies the dynamics of latent variables as they change over time. Moreover, $\eta_{t}$ and $\varepsilon_{t}$ were independently and normally distributed error terms, and $Z_{t}$, $T_{t}$, and $R_{t}$ were model matrices mixed with known values (0 or 1) and other unknown parameters [24].

The spike-and-slab prior was applied for the optimal variable selection, and the Kalman filter was used for time-series prediction [24]. We used the Markov chain Monte Carlo algorithm with 10,000 iterations to simulate the posterior distribution of predictions, and then, Bayesian model averaging was used to smooth over a large number of potential results. The model fitting goodness was evaluated by root mean square error, mean absolute percentage error, and *R*^2^ [25].

Subsequently, we used the fitted model to predict the EV71-associated HFMD cases from 2017 to 2019. The relative difference (RD) between predicted and observed cases in the BSTS model was used to estimate the EV71 vaccination effectiveness. The RD was calculated by subtracting the observed cases from the predicted cases and then dividing by the predicted cases. Additionally, we performed age-stratified analyses to evaluate the effectiveness of EV71 vaccination program among children aged 0 to 2, 3 to 4, and 5 to 14 years.

### Ethical Considerations

The study received ethical exemption approval from the Ethics Committee of Jiangsu Provincial Center for Disease Control and Prevention (SL2025-B031-01), and the requirement for informed consent was waived by the ethics committee. The HFMD surveillance data used in this study were collected as part of legally mandated infectious disease prevention and control activities. These data were obtained from compliant sources and stored securely, and their secondary use fully adhered to ethical standards. All analyses were performed on deidentified datasets, ensuring that no harm or infringement of rights occurred to data providers. Given that the study is beneficial to HFMD prevention and control and presents minimal risk, it meets the criteria for exemption from ethical review.

## Results

### HFMD Cases

This analysis included 932,274 cases of HFMD in Jiangsu during the period from 2012 to 2019, which exhibited seasonal and biannual patterns (Figure 1B). Among the reported cases, 5190 (0.56%) were classified as severe (Figure 1C), and an estimated 140,876 (15.11%) cases were attributed to EV71.

Notably, we observed that the proportion of EV71 cases declined sharply after the vaccine implementation, with estimated case counts of 22,303 in 2017, 9796 in 2018, and 3900 in 2019. For instance, the number of EV71 cases peaked at nearly 8000 in May of 2012, whereas in 2019, it was almost below 1100 (Figure 1D).

### The Fitting Goodness of BSTS Models

On the basis of the training sets, we constructed the BSTS model and fitted the monthly cases from 2012 to 2016 (Multimedia Appendix 1). In summary, we observed that the performance of the model was accurate and robust, with a root mean square error of 0.33, a mean absolute percentage error of 3.46%, and an *R*^2^ of 78.76%.

### The Effectiveness of the EV71 Vaccination Program During 2017 to 2019

**Figure 2.**
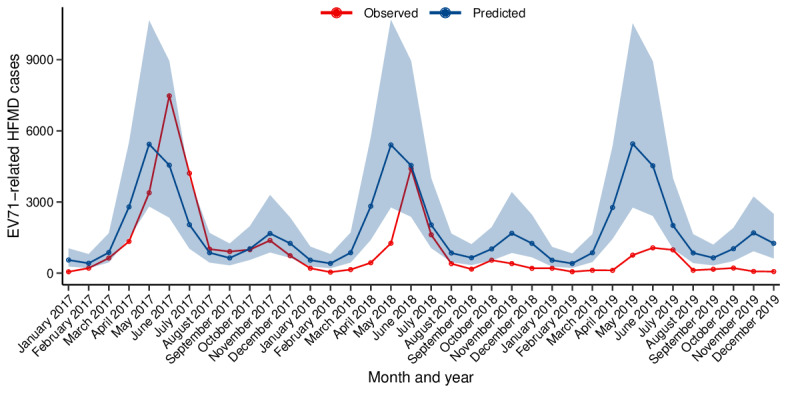
Monthly trends of predicted counterfactual enterovirus A71 (EV71)–related cases (blue) and observed cases (red) in Jiangsu Province during 2017 to 2019. HFMD: hand, foot, and mouth disease.

Overall, during the EV71 vaccination period (2017 to 2019), the observed number of EV71 cases was significantly lower than the number forecasted by the model (Figure 2 and Table 1). We estimated that 30,117 (95% credible interval −1777 to 93,601) EV71 cases were prevented by EV71 vaccination, corresponding to a relative reduction of 45.55% (95% credible interval −5.19% to 72.22%).

**Table 1.** The observed, predicted, and prevented enterovirus A71–induced hand, foot, and mouth disease cases and relative reductions among children in Jiangsu Province, 2017 to 2019.

Year	Observed cases, n	Predicted cases, n (95% credible interval)	Prevented cases^a^, n (95% credible interval)	Relative reduction^b^ (%; 95% credible interval)
2017‐2019	35,999	66,116 (34,222 to 129,600)	30,117 (−1777 to 93,601)	45.55 (−5.19 to 72.22)
2017	22,303	22,077 (11,446 to 43,131)	−226 (−10,857 to 20,828)	−1.03 (−94.85 to 48.29)
2018	9796	22,032 (11,382 to 43,651)	12,236 (1586 to 33,855)	55.54 (13.94 to 77.56)
2019	3900	22,008 (11,394 to 42,818)	18,108 (7494 to 38,918)	82.28 (65.77 to 90.89)

a Prevented cases were calculated as predicted cases under the counterfactual scenario without vaccination minus observed cases.

b Relative reduction values were calculated by dividing the number of prevented cases by the number of predicted cases.

Furthermore, the magnitude of the decline exhibited a progressive increase over years. We observed that −226 (95% credible interval −10,857 to 20,828) EV71-infected cases were prevented in 2017, whereas the number of prevented cases increased to 12,236 (95% credible interval 1586-33,855) in 2018 and further to 18,108 (95% credible interval 7494-38,918) in 2019, with the relative reduction of −1.03% (95% credible interval −94.85% to 48.29%), 55.54% (95% credible interval 13.94%-77.56%), and 82.28% (95% credible interval 65.77%-90.89%) for the respective years.

### The Effectiveness of the EV71 Vaccination Program by Age Group

**Figure 3.**
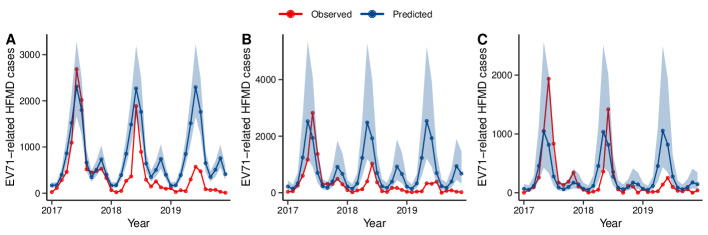
Monthly trends of predicted counterfactual enterovirus A71 (EV71)–related cases (blue) and observed cases (red) among different age groups in Jiangsu Province during 2017 to 2019. HFMD: hand, foot, and mouth disease. (A) Children aged 0-2 years; (B) children aged 3-4 years; (C) children older than 4 years.

The relative reductions of EV71-related HFMD cases by age group from 2017 to 2019 are shown in Figure 3 and Table 2. Among age subgroups, the RD exhibited an increasing trend over the years, with the highest RD observed in the children aged 3 to 4 years. The relative reduction during 2017 to 2019 was 48.09% (95% credible interval 28.00%-62.98%) for children aged 0 to 2 years, 57.68% (95% credible interval 13.04%-79.65%) for children aged 3 to 4 years, and 16.75% (95% credible interval −96.53% to 65.47%) for children older than 4 years.

**Table 2.** Age-stratified relative reduction of enterovirus A71–induced hand, foot, and mouth disease cases among children in Jiangsu Province, 2017 to 2019.

Age group (years)	Relative reduction in 2017‐2019, (%; 95% credible interval)	Relative reduction in 2017, (%; 95% credible interval)	Relative reduction in 2018, (%; 95% credible interval)	Relative reduction in 2019 (%; 95% credible interval)
0-2	48.09 (28.00 to 62.98)	9.16 (−25.83 to 35.23)	54.20 (36.43 to 67.48)	81.20 (73.91 to 86.53)
3-4	57.68 (13.04 to 79.65)	15.70% (−72.40 to 59.97)	72.03 (42.94 to 86.55)	85.44 (69.72 to 92.91)
>4	16.75 (−96.53 to 65.47)	−52.51 (−260.67 to 7.33)	24.82 (−76.16 to 69.09)	77.80 (47.28 to 90.62)

## Discussion

### Principal Findings

In this study, we used an advanced BSTS model to conduct the first population-based assessment of EV71 vaccine effectiveness using real-world surveillance data from Jiangsu Province, China. Our analysis revealed a stable and consistent seasonal fluctuation in EV71 cases from 2012 to 2016. However, a progressive decline in case numbers over subsequent years was observed from 2017 to 2019. On the basis of a counterfactual analysis framework, we estimated that the EV71 vaccination program led to a 45.55% reduction in EV71-associated HFMD cases. Furthermore, we identified age-related differences in EV71 vaccine effectiveness, with varying levels of protection observed across different age groups. These findings provide robust empirical evidence supporting the real-world effectiveness of the EV71 vaccination program in Jiangsu Province and offer valuable data to inform future public health strategies targeting EV71-related HFMD.

Although EV71 cases declined after vaccine introduction in 2017, the overall HFMD cases showed a temporary increase in 2018. This may be explained by intensified surveillance and reporting efforts following vaccine rollout, as well as the circulation of non-EV71 enterovirus serotypes during that year. Similar postvaccination increases in total HFMD notifications have also been reported in other provinces [16,17,26,27]. Given the geographical proximity and demographic similarities between Jiangsu and Zhejiang Provinces, our findings demonstrate remarkable consistency with the results reported by Zheng et al [17] in Zhejiang, further supporting the robustness of our findings.

However, the protective efficacy observed in our study was lower than that reported in Guangdong Province and particularly in Chengdu City of Sichuan Province, where vaccine effectiveness was estimated to be as high as 60% during 2017 to 2018 [18]. These regional differences may be attributable to variations in age distribution and, more importantly, vaccination coverage rates [17]. For instance, Guangdong Province achieved a median coverage rate of 35.8% in 2019 [16], whereas a study conducted in Chengdu City among children aged 6 to 59 months reported a coverage rate of 54.3% in 2018 [18]. In contrast, Jiangsu Province reported markedly lower vaccination coverage rates. In 2019, vaccination coverage was only 10.6% in Nanjing, the provincial capital, and an estimated 12.7% in Lianyungang [28,29], underscoring the suboptimal vaccination uptake across Jiangsu Province.

Our longitudinal analysis also demonstrated a progressive increase in the relative reduction rate of EV71 cases, which may reflect the incremental improvements in vaccine coverage in Jiangsu Province. For example, in Nanjing, the coverage rate rose from 5.7% in 2017 to 12.6% in 2018, before slightly declining to 10.6% in 2019. Additionally, we found that the vaccine effectiveness was highest among children aged 3 to 4 years, compared with those younger than 3 years or older than 4 years, during the 2017 to 2019 period. This age-specific trend aligns with findings from previous studies, yet it failed to attain statistical significance [17,30]. The superior benefit observed in children aged 3 to 4 years is likely attributable to their more mature immune systems compared with younger children, enabling a stronger and more durable vaccine-induced immune response [31]. In contrast, among children older than 4 years, a substantial proportion may have already acquired natural immunity through prior EV71 infection, thereby reducing the incremental benefit of vaccination. Additionally, differences in vaccination coverage across age groups may also have contributed to the observed pattern.

Several limitations of this study should be acknowledged. First, our findings may be susceptible to unmeasured confounding factors, such as socioeconomic conditions, differences in vaccination coverage across age groups, or concurrent changes in health care policy. However, the consistency of our results with prior studies, suggesting no substantial unmeasured confounding, strengthens confidence in the robustness of these estimates of EV71 vaccination effectiveness. Second, as an ecological study, we evaluated vaccine effectiveness at the population level and lacked access to individual-level data, which would be necessary to account for potential confounding variables and assess individual-level determinants of vaccine response. Third, although estimates based on EV71-positive rates may involve uncertainty due to incomplete testing, the routine surveillance sampling approach supports their validity. Finally, as with other similar evaluations, our study was limited by the relatively short postvaccination observation period. The wide credible intervals indicate substantial uncertainty in the estimated number of cases prevented. Ongoing long-term surveillance will be essential to fully understand the durability and long-term effectiveness of the EV71 vaccination program.

### Conclusions

Our study provides a robust population-level evaluation of the effect of the EV71 vaccination program in Jiangsu Province during the period of 2017 to 2019. We observed a substantial reduction in EV71-associated HFMD cases following the implementation of the vaccination policy, with the magnitude of the decline increasing progressively in line with improvements in vaccine coverage. Additionally, vaccine effectiveness was notably higher among children aged 3 to 4 years compared with other age groups. These findings provide strong real-world evidence supporting the utility of the EV71 vaccination program in reducing disease burden and offer a scientific basis for optimizing population-level intervention strategies to control EV71-related HFMD.

## Supplementary material

10.2196/85604 Multimedia Appendix 1 Fitting performance of the Bayesian structural time series model for monthly cases from 2012 to 2016.

10.2196/85604 Checklist 1 STROBE checklist for observational studies.

## Abbreviations

BSTS: Bayesian structural time series
EV71: enterovirus A71
HFMD: hand, foot, and mouth disease
RD: relative difference
STROBE: Strengthening the Reporting of Observational Studies in Epidemiology
