# Supplementary figures and images for: Population-Level Impact of the Enterovirus A71 Vaccination Program on Hand, Foot, and Mouth Disease: Ecological Time-Series Study
Source: JMIR Public Health Surveill. 2026 Mar 10;12:e85604. doi: 10.2196/85604 (PMC12975000; doi:10.2196/85604)

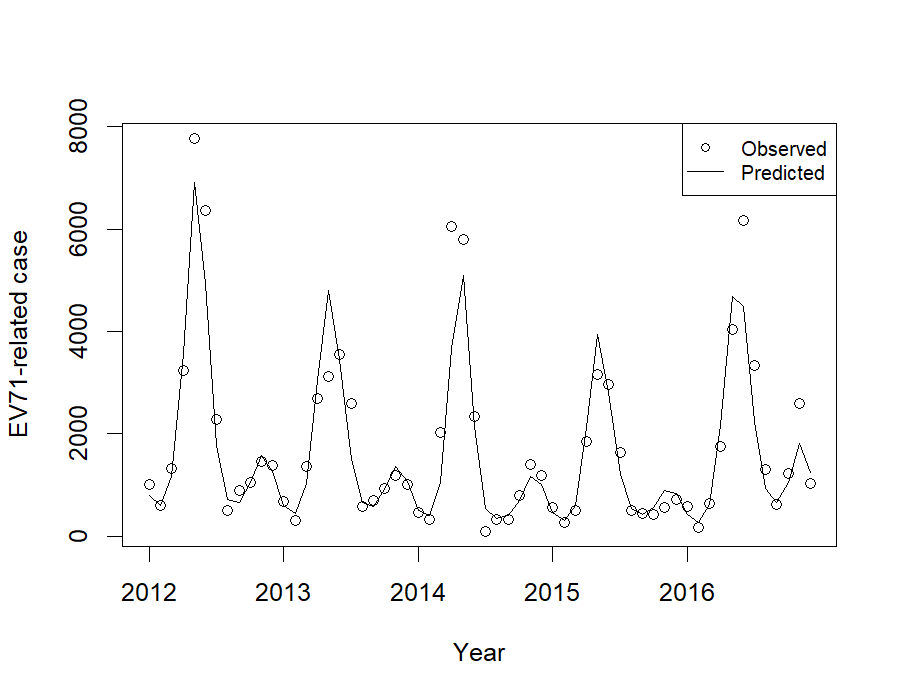

Supplement: Multimedia Appendix 1 [file publichealth-v12-e85604-s001.png]
