# Supplementary material for: Population-Level Impact of the Enterovirus A71 Vaccination Program on Hand, Foot, and Mouth Disease: Ecological Time-Series Study
Source: JMIR Public Health Surveill. 2026 Mar 10;12:e85604. doi: 10.2196/85604 (PMC12975000; doi:10.2196/85604)
Supplement: Checklist 1 [file publichealth-v12-e85604-s002.pdf]

The STROBE reporting checklist

For checking that observational epidemiology research articles can be understood and used by everyone

> Note
>
> If you have not used a reporting guideline before, read about how and why to use them and check whether STROBE is the most applicable reporting guideline for your work.
>
> Reporting guidelines are most useful when used early in research. When writing a manuscript or application, consider using the Full Guidance where you'll see explanations and examples for each item.
>
> After writing, demonstrate adherence by completing this checklist:
>
> 1. Specify where each item is described (see Note 1).
> 2. Cite this checklist (See Note 2).
> 3. Include your completed checklist as a supplement when submitting to a journal so that future readers can use it to find information.

	Item Description	Location (or reason for not reporting)
**Title and abstract**		
1a. Indicate the study's design	Indicate the study's design with a commonly used term in the title or the abstract.	Page 1, Title
1b. Abstract	Provide in the abstract an informative and balanced summary of what was done and what was found.	Page 1-2, Abstract
**Introduction**		
2. Background / rationale	Explain the scientific background and rationale for the investigation being reported.	Page 3, Introduction, para. 1-2
3. Objectives	State specific objectives, including any prespecified hypotheses.	Page 4, Introduction, paragraph 5
**Methods**		
4. Study design	Present key elements of study design early in the paper.	Page 4, Methods, para. 1
5. Setting	Describe the setting, locations, and relevant dates, including periods of recruitment, exposure, follow-up, and data collection.	Page 4, Methods, para. 1-3
6a. Eligibility criteria	**Cohort study:** Give the eligibility criteria, and the sources and methods of selection of participants. Describe methods of follow-up. **Case-control study:** Give the eligibility criteria, and the sources and	Page 5, Methods, para. 2<br>All HFMD cases aged 0–14 years reported in Jiangsu Province during 2017–2019

	methods of case ascertainment and control selection. Give the rationale for the choice of cases and controls. **Cross-sectional study:** Give the eligibility criteria, and the sources and methods of selection of participants.	were included in the analysis because HFMD is a nationally notifiable disease. As this study used complete population-based surveillance data, no additional sampling or follow-up procedures were required.
6b. Matching criteria	**Cohort study:** For matched studies, give matching criteria and number of exposed and unexposed. **Case-control study:** For matched studies, give matching criteria and the number of controls per case.	Not Applicable
7. Variables	Clearly define all outcomes, exposures, predictors, potential confounders, and effect modifiers. Give diagnostic criteria, if applicable.	Page 5, Methods, para. 2-3; Page 6, Statistical analysis, para. 3<br>The primary outcome was the number of EV71-associated HFMD cases, estimated using the age-specific EV71 positivity rate multiplied by the total number of reported HFMD cases. The exposure of interest was the implementation of the EV71 vaccination program beginning in 2017. No individual-level predictors, confounders, or effect modifiers were included because the analysis was based on aggregated surveillance data rather than individual-level assessments.
8. Data sources / measurement	For each variable of interest give sources of data and details of methods of assessment (measurement). Describe comparability of assessment methods if there is more than one group.	All variables were derived from the National Notifiable Disease Surveillance System (NNDRS). HFMD cases were diagnosed and reported by healthcare facilities according to the national case definitions and reporting guidelines. EV71 detection was based on routine laboratory surveillance, using standardized PCR testing protocols across all participating laboratories. Because all data were

		obtained from the same surveillance platform with unified diagnostic and reporting procedures, assessment methods were comparable across all groups.
9. Bias	Describe any efforts to address potential sources of bias.	Page 5, Statistical analysis, para. 1<br>Potential sources of bias were minimized by using province-wide, mandatory notifiable disease surveillance data that cover all health facilities in Jiangsu. Case definitions and laboratory testing followed uniform national guidelines, ensuring consistency over time. Additionally, the BSTS model accounted for long-term trends and seasonality, reducing bias arising from temporal fluctuations unrelated to vaccination. Since the analysis was based on aggregated surveillance data, individual-level selection or information bias was not applicable.
10. Study size	Explain how the study size was arrived at.	Page 4, Methods, para. 1
11. Quantitative variables	Explain how quantitative variables were handled in the analyses. If applicable, describe which groupings were chosen, and why.	Page 5, Methods, para. 5<br>Age was treated as a categorical variable in the analysis, grouped as 0–2, 3–4, and 5–14 years to reflect meaningful pediatric age ranges and potential differences in vaccine impact. HFMD cases were analyzed as counts per month for temporal trend analyses.
12a. Statistical methods	Describe all statistical methods, including those used to control for confounding.	Page 5-6, Statistical analysis
12b. Statistical methods – subgroups and interactions	Describe any methods used to examine subgroups and interactions.	Page 6, Statistical analysis, Methods, para. 5

12c. Statistical methods – missing data	Explain how missing data were addressed.	Page 5, Methods, para. 4
12di. Statistical methods – loss to follow-up	**Cohort study:** If applicable, describe how loss to follow-up was addressed.	Not Applicable
12dii. Statistical methods – matching cases and controls	**Case-control study:** If applicable, explain how matching of cases and controls was addressed.	Not Applicable
12diii. Statistical methods – sampling strategy	**Cross-sectional study:** If applicable, describe analytical methods taking account of sampling strategy.	Not applicable. This study did not involve sampling because HFMD is a nationally notifiable infectious disease in China, and all HFMD cases aged 0–14 years reported in Jiangsu Province during 2017–2019 were included in the analysis.
12e. Statistical methods – sensitivity analyses	Describe any sensitivity analyses.	Not applicable. This study did not involve multiple model specifications or alternative assumptions; therefore, no sensitivity analyses were required or conducted.
**Results**		
13a. Participant numbers	Report the numbers of individuals at each stage of the study—e.g., numbers potentially eligible, examined for eligibility, confirmed eligible, included in the study, completing follow-up, and analysed; Consider use of a flow diagram.	Not applicable. This study used complete surveillance data, and all reported HFMD cases aged 0–14 years in Jiangsu Province during 2017–2019 were included; therefore, no eligibility screening or flow diagram was required.
13b. Participants – non-participation	Give reasons for non-participation at each stage.	Not applicable. This study used a complete population-based surveillance dataset, so there was no participant recruitment or follow-up process, there were no instances of non-participation to be reported.
13c. Participants – flow diagram	Consider use of a flow diagram.	Not applicable. All eligible HFMD cases aged 0–14 years in Jiangsu Province during 2017–2019 were included

		based on mandatory notifiable disease reporting. As no screening or enrollment procedures occurred, a participant flow diagram is not applicable.
14a. Descriptive data – participant characteristics	Give characteristics of study participants (e.g., demographic, clinical, social) and information on exposures and potential confounders. Present the information in a table.	Not applicable. This ecological study used aggregated case counts only, without access to individual-level demographic, clinical, or exposure information; therefore, participant characteristics and potential confounders could not be described.
14b. Descriptive data – missing data	Indicate the number of participants with missing data for each variable of interest.	Not applicable. Because the analysis was based on aggregated surveillance indicators rather than individual-level data, the concept of missing data for each variable does not apply.
14c. Descriptive data – follow-up time	**Cohort study:** Summarise follow-up time—e.g., average and total amount.	Not applicable.
15. Outcome data	**Cohort study:** Report numbers of outcome events or summary measures over time. **Case-control study:** Report numbers in each exposure category, or summary measures of exposure. **Cross-sectional study:** Report numbers of outcome events or summary measures.	Page 7, Results, para. 1-2
16a. Main results	Give unadjusted estimates and, if applicable, confounder-adjusted estimates and their precision (e.g., 95% confidence intervals). Make clear which confounders were adjusted for and why they were included.	Page 7, Results, para. 4
16b. Main results – category boundaries	Report category boundaries when continuous variables were categorised.	Age, originally recorded as a continuous variable, was categorized into three groups (0–2, 3–4, and 5–14 years) for stratified analyses. These boundaries were selected based on epidemiological patterns of HFMD susceptibility and vaccination strategy considerations.

16c. Main results – risk	If relevant, consider translating estimates of relative risk into absolute risk for a meaningful time period.	Not applicable. This study did not estimate individual-level absolute or relative risks; instead, it evaluated population-level reductions in EV71-associated HFMD cases using an ecological impact assessment framework.
17. Other analyses	Report other analyses done—e.g., analyses of subgroups and interactions, and sensitivity analyses.	Page 8, Results, Table. 2
**Discussion**		
18. Key results	Summarise key results with reference to study objectives.	Page 8, Discussion, para. 1
19. Limitations	Discuss limitations of the study, taking into account sources of potential bias or imprecision. Discuss both direction and magnitude of any potential bias.	Page 9, Discussion, para. 5
20. Interpretation	Give a cautious overall interpretation considering objectives, limitations, multiplicity of analyses, results from similar studies, and other relevant evidence.	Page 10, Conclusion
21. Generalisability	Discuss the generalisability (external validity) of the study results.	Page 9, Discussion, para. 2-3
**Other information**		
22. Funding	Give the source of funding and the role of the funders for the present study and, if applicable, for the original study on which the present article is based.	Page 11, Funding Statement

1 How to specify where content is

Tell the reader where they can find information. E.g.,

- Results; paragraph 2
- Methods, Participants; paragraphs 1 & 2.
- Table 3
- Supplement B, para. 4

If you have chosen not to describe an item, explain why. You can do this in the checklist, or as a note below it.

You can describe items in the article body, or in tables, figures, or supplementary materials, and should prioritize items you feel are most important to your intended audience. The order of items in your manuscript does not need to match the order of items in this checklist. You can decide how best to structure your work.

2 How to cite

Describe how you used STROBE at the end of your Methods section, referencing the resources you used e.g.,

> ‘We used the STROBE reporting guideline(1) to draft this manuscript, and the STROBE reporting checklist(2) when editing, included in supplement A’

If you use a reporting checklist, remember to include it as a supplement when publishing so that readers can easily find information and see how you have interpreted the guidance.

1. Elm E von, Altman DG, Egger M, Pocock SJ, Gøtzsche PC, Vandenbroucke JP, et al. The strengthening the reporting of observational studies in epidemiology (STROBE) statement: Guidelines for reporting observational studies. Annals of Internal Medicine [Internet]. 2007 Oct;147(8):573–7. Available from: https://www.acpjournals.org/doi/10.7326/0003-4819-147-8-200710160-00010

2. Elm E von, Altman DG, Egger M, Pocock SJ, Gøtzsche PC, Vandenbroucke JP, et al. The STROBE reporting checklist. In: Harwood J, Albury C, Beyer J de, Schlüssel M, Collins G, editors. The EQUATOR network reporting guideline platform [Internet]. The UK EQUATOR Centre; 2025. Available from: https:/resources.equator-network.org/reporting-guidelines/strobe/strobe-checklist.docx
